# Systemic Exposure to PAHs and Benzene in Firefighters Suppressing Controlled Structure Fires

**DOI:** 10.1093/annhyg/meu036

**Published:** 2014-06-06

**Authors:** Kenneth W. Fent, Judith Eisenberg, John Snawder, Deborah Sammons, Joachim D. Pleil, Matthew A. Stiegel, Charles Mueller, Gavin P. Horn, James Dalton

**Affiliations:** 1.Division of Surveillance, Hazard Evaluations, and Field Studies, National Institute for Occupational Safety and Health, 4676 Columbia Parkway, Cincinnati, OH 45226, USA; 2.Division of Applied Research and Technology, National Institute for Occupational Safety and Health, 4676 Columbia Parkway, Cincinnati, OH 45226, USA; 3.Human Exposure and Atmospheric Sciences Division, U.S. Environmental Protection Agency, 109 T.W. Alexander Drive, Research Triangle Park, NC 27709, USA; 4.Oak Ridge Institute for Science and Education/U.S. Environmental Protection Agency Research Fellow, Gillings School of Global Public Health, University of North Carolina, Chapel Hill, NC 27599, USA; 5.Illinois Fire Service Institute, University of Illinois-Urbana/Champaign, 11 Gerty Drive, Champaign, IL 61820, USA; 6.Research and Development Section, Training Division, Chicago Fire Department, 558 West DeKoven Street, Chicago, IL 60607, USA

**Keywords:** aromatic hydrocarbons, benzene, biomarkers, dermal exposure, exhaled breath, firefighters, PAHs, urine

## Abstract

Turnout gear provides protection against dermal exposure to contaminants during firefighting; however, the level of protection is unknown. We explored the dermal contribution to the systemic dose of polycyclic aromatic hydrocarbons (PAHs) and other aromatic hydrocarbons in firefighters during suppression and overhaul of controlled structure burns. The study was organized into two rounds, three controlled burns per round, and five firefighters per burn. The firefighters wore new or laundered turnout gear tested before each burn to ensure lack of PAH contamination. To ensure that any increase in systemic PAH levels after the burn was the result of dermal rather than inhalation exposure, the firefighters did not remove their self-contained breathing apparatus until overhaul was completed and they were >30 m upwind from the burn structure. Specimens were collected before and at intervals after the burn for biomarker analysis. Urine was analyzed for phenanthrene equivalents using enzyme-linked immunosorbent assay and a benzene metabolite (s-phenylmercapturic acid) using liquid chromatography/tandem mass spectrometry; both were adjusted by creatinine. Exhaled breath collected on thermal desorption tubes was analyzed for PAHs and other aromatic hydrocarbons using gas chromatography/mass spectrometry. We collected personal air samples during the burn and skin wipe samples (corn oil medium) on several body sites before and after the burn. The air and wipe samples were analyzed for PAHs using a liquid chromatography with photodiode array detection. We explored possible changes in external exposures or biomarkers over time and the relationships between these variables using non-parametric sign tests and Spearman tests, respectively. We found significantly elevated (P < 0.05) post-exposure breath concentrations of benzene compared with pre-exposure concentrations for both rounds. We also found significantly elevated post-exposure levels of PAHs on the neck compared with pre-exposure levels for round 1. We found statistically significant positive correlations between external exposures (i.e. personal air concentrations of PAHs) and biomarkers (i.e. change in urinary PAH metabolite levels in round 1 and change in breath concentrations of benzene in round 2). The results suggest that firefighters wearing full protective ensembles absorbed combustion products into their bodies. The PAHs most likely entered firefighters’ bodies through their skin, with the neck being the primary site of exposure and absorption due to the lower level of dermal protection afforded by hoods. Aromatic hydrocarbons could have been absorbed dermally during firefighting or inhaled during the doffing of gear that was off-gassing contaminants.

## INTRODUCTION

The 346 000 career firefighters and 783 000 volunteer firefighters in the USA (NFPA, 2013a) are potentially exposed to a variety of different chemicals during fire suppression. Polycyclic aromatic hydrocarbons (PAHs) are components of incomplete combustion that can exist in both particle and gas phase. Of the 18 PAHs that are commonly produced during fires, the International Agency for Research on Cancer (IARC) classified benzo[a]pyrene as carcinogenic to humans (Group 1) and eight others as probably or possibly carcinogenic to humans (Group 2A or 2B) (IARC, 2002 , 2010). In addition to PAHs, nearly all fires will produce other potentially carcinogenic aromatic hydrocarbons such as a benzene (IARC, 2012).

When firefighters suppress structure fires, they typically wear National Fire Protection Association (NFPA) 1971/1981 compliant protective ensembles (NFPA, 2013b, c). These ensembles include a self-contained breathing apparatus (SCBA), which has the highest assigned protection factor (10 000) of any respirator (29 CFR 1910.134). Burgess and Crutchfield (1995) found that firefighters under high physical exertion (walking on an inclined treadmill) could overbreathe their SCBA; yet most SCBA in that study still provided protection factors > 10 000. At lower exertion levels, SCBA should virtually eliminate inhalation exposures to combustion products like PAHs and benzene by maintaining positive pressure inside the face mask at all times.

The degree of dermal protection to combustion products afforded by protective ensembles is currently unknown. To date, only a few studies have explored dermal exposure and absorption of combustion products in firefighters. Investigators at the Queensland Fire and Rescue Service (QFRS) found that aromatic hydrocarbons may penetrate turnout gear and contact skin (QFRS, 2011a,b) although the methods used could have created a gradient that pulled contaminants into the gear. Caux *et al.* (2002) and Laitinen *et al.* (2010) found that firefighters wearing full protective ensembles may absorb PAHs and/or benzene during firefighting. However, inhalation exposures to environmental smoke from premature removal of SCBA and transfer of PAHs from contaminated gear to the skin were possible in these studies.

Our hypothesis was that despite wearing full protective ensembles, firefighters absorb PAHs and other aromatic hydrocarbons through their skin during firefighting. The absorption of these compounds may be shown by an increase in their biological levels following the exposure period. To address the aforementioned limitations, firefighters participating in our study wore laundered turnout gear and did not remove their SCBA until the fire was completely extinguished, and they were a specified distance away from the burn structure. A summary report from this study was provided to the participants and posted on the National Institute for Occupational Safety and Health (NIOSH) website according to our regulations and policy.

## METHODS

### Recruitment of firefighters

Inclusion criteria for this study were non-smoking males 45 years of age or younger who were instructors with the Chicago Fire Department. The Coordinator of Research and Development at the Chicago Fire Department distributed our study information sheet to eligible Chicago firefighters. After receiving volunteers, he coordinated with the fire chiefs from each station to schedule five firefighter participants for each day of the study. Participants were instructed to not eat char-grilled foods and avoid second-hand tobacco smoke for 2 days prior to the start of the study. Scheduling was also done to ensure that the participants had at least one day off from firefighting activities before reporting to the study site.

Round 1 of the study was in August 2010; round 2 was 1 year later. Firefighters were consented before each round. Fifteen firefighters participated in each round (five firefighters each day). Twelve firefighters from round 1 repeated the study during round 2. Each round consisted of three controlled structure burns (one burn each day).

### Study design

This study was conducted at the University of Illinois Fire Service Institute training facility. We had five sample collection periods: pre-exposure (~1h before the controlled burn), exposure (during the controlled burn), post-exposure (10–40min after the controlled burn), 3h after the controlled burn, and 6h after the controlled burn. The timing of the samples was based in part on previous studies involving firefighters that found maximum excretion of PAHs 4–8h after exposure (Caux *et al.*, 2002; Laitinen *et al.*, 2010) and rapid excretion of benzene (Laitinen *et al.*, 2010).

Table 1 summarizes the controlled burns for each round, with some important differences between these rounds noted. Prior to each day of the study, a burn room and target room were constructed of drywall inside these structures. These rooms were connected to each other by an open doorway. Fuel packages typical of family room furniture were placed inside the burn room. Thermocouples were placed at various heights inside the target and burn rooms.

**Table 1.** Summary of controlled burns for each round of the study with important differences noted

Round/fire scenario	Day/burn	Exposure times by response phase (min)			
		Active fire	Knockdown	Overhaul	Total
1. Timber-framed structure, drywall interior, 33 m^3^ burn room, 33 m^3^ target room, firefighters were mostly stationary, hoods shorter than in round 2, exercises involving wood smoke also took place at the training facility	1	10	1	4	15
	2	11	3	16	30
	3	15	7	7	29
2. Intermodal metal container, drywall interior, 15 m^3^ burn room, 35 m^3^ target room, firefighters were mobile and rotated positions (except for nozzleman and company officer)	1	10	2	8	20
	2	10	3	5	18
	3	10	4	4	18

Fires were started by the interior safety officer by igniting newspapers inside a wastebasket. Firefighters were inside the target room during active fire (when the fires grew from the ignition stage to the fully developed stage) and knockdown (when the nozzleman suppressed the fires with the hosestream) and then moved into the burn room for overhaul (when the firefighters searched for and extinguished any residual flames). Water was applied to the burn room to control the fire size if ceiling temperatures inside the burn room exceeded 425°C or 1.2-m height temperatures inside the target room exceeded 120°C or if the company officer/safety officer felt it necessary for crew safety. Firefighters were instructed to suppress the fires within 15min after ignition.

The period of active fire was intended to simulate the time period encompassing a typical interior attack when firefighters would be exposed to products of combustion. For round 1, firefighters stood or crouched in the target room during this period. For round 2, the firefighters (except for the nozzleman and company officer who stood or crouched in attack position with a charged hoseline) rotated between three stations in the target room that were intended to simulate searching for victims, pulling down a ceiling to expose overhead fires (Fig. 1), and resting at the back of the structure.

**1:**
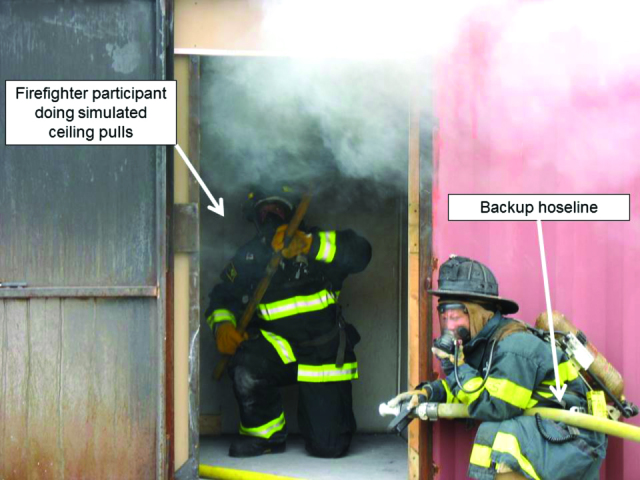
Firefighter participant conducting simulated ceiling pull task during a round 2 controlled burn inside an intermodal metal container.

The firefighters wore protective ensembles that complied with the 2007 editions of the NFPA 1971 and 1981 standards that were applicable at the time of this study (NFPA, 2007a,b). The gloves and turnout gear were laundered before use. The hoods used in round 2 were brand new and ~7.5cm longer than the laundered hoods used in round 1. We tested the turnout gear before each burn to ensure that the gear had minimal PAH contamination. Participants did not remove their SCBA until overhaul was completed, and they were at least 30 m northwest of the burn structure. Prevailing winds were out of the southwest. Following each burn, we instructed the participants to stay inside a climate-controlled building for the rest of the day (6h) to minimize any potential exposures from other training exercises taking place at the facility. During round 1 only, training exercises involving wood smoke were performed at the facility several hours after our study’s controlled burns.

### Air sampling

We conducted personal air sampling for PAHs during the controlled burns (from ignition to completion of overhaul). We sampled respirable particles using an aluminum cyclone and polytetrafluoroethylene filter (SKC Inc., Eighty Four, PA, USA) and gases and vapors using an attached SKC XAD-2 sorbent tube. The flow rate we used (2.5 l min^−1^) provides a 4-µm aerodynamic diameter cut point for the cyclone. To prevent fire damage to the sampling train, we wrapped it in Nomex® flame-resistant material (DuPont, Wilmington, DE, USA) and replaced the cyclone’s plastic grit pot with a fabricated aluminum grit pot. The sampling pump and majority of the tubing were positioned on the interior of the turnout gear.

Of the 30 personal sampling pumps used in this study, only 2 functioned properly through the completion of the exercises. Three pumps had disconnected tubing and 25 faulted before completion of overhaul. Of the 25 that faulted, 12 stopped working before knockdown. The median amount of time the pumps ran after fire ignition was 15min, ranging from 2 to 24min. For the 12 pumps that faulted before knockdown, air concentrations were calculated based on the amount of time the pumps ran during active fire; we assumed that the PAH air concentrations during active fire were fairly constant over time. For the 15 pumps that continued to run after knockdown, air concentrations were calculated based on the total time period of active fire (see Table 1); we assumed that the PAH air concentrations after knockdown were negligible. These calculations were done to provide estimates of airborne PAH exposures during the most contaminated time period of active fire.

NIOSH Method 5506 (NIOSH, 2013a) was used to analyze these air samples, which employs high-performance liquid chromatography with photodiode array detection (HPLC-PDA). This method is able to differentiate between 17 PAHs. However, for this article, we report air concentrations of total PAHs. The calculation for total PAHs accounts for all peaks in the PAH response region of the chromatogram; we combined these results for both the particulate and gas phase.

### Dermal wipe sampling

Wipe sampling was used to measure dermal exposure to PAHs on the forearms, hands, neck, face, and scrotum. Samples of both forearms and the neck and scrotum were collected pre- and post-exposure. Samples of both hands and the face were only collected post-exposure. We sprayed corn oil on skin using spray bottles to facilitate the collection of lipophilic PAHs. Four to six sprays (~0.5ml per spray) were applied evenly depending on the surface area of the skin site and then wiped off using AlphaWipes® (TX1004, Texwipe®, Kernersville, NC, USA). For the scrotal wipe samples, wipes impregnated with 1ml of corn oil and nitrile gloves were given to the firefighters with instructions on how to collect the samples. This is similar to the technique used by Väänänen *et al.* (2005). The wipe samples were analyzed by NIOSH Method 5506 (NIOSH, 2013a), which employs HPLC-PDA.

The corn oil that we used for the dermal wipe samples had a complex chemical matrix. Several peaks were present in the media blanks (wipes impregnated with 2ml of corn oil) in the region of the chromatogram where the PAHs would typically elute, so we could not use total PAHs as a metric for the dermal wipe samples. Instead, we selected six PAHs (anthracene, benzo[a]pyrene, chrysene, fluoranthene, phenanthrene, and pyrene) to sum as a surrogate of the total PAHs. The PAHs we selected had the highest rates of detection and mostly eluted in the region outside of the interfering peaks. They also corresponded with the PAHs toward which the urine enzyme-linked immunosorbent assay (ELISA) method had the greatest sensitivity. The analytical results were blank corrected.

Dermal exposure levels of PAHs were standardized by the surface area of the skin collection site. The surface areas of the forearms (0.15 m^2^) and hands (0.11 m^2^) were based on mean dermal exposure factor data for adult males (U.S. Environmental Protection Agency [EPA], 2011). We estimated surface areas of the face (0.068 m^2^) and scrotum (0.054 m^2^) by dividing the surface areas of the head and hand, respectively, by two. The surface area of the neck (0.042 m^2^) was determined based on data from Lund and Browder (1944) showing the neck accounts for 2% of the total body surface area, which is 2.1 m^2^ for adult males 30–39 years of age (EPA, 2011).

### Exhaled breath sampling

Exhaled breath can be used to assess volatile fraction of the systemic uptake, regardless of route of entry (presumably dermal in this case) (Pleil and Lindstrom, 1998; Pleil, 2008). We measured the concentrations of combustion products in exhaled breath samples collected pre-, post-, and 6-h post-exposure. The firefighters were instructed to take a deep breath in and then forcefully exhale into the Bio-VOC™ sampler (Markes International, Wilmington, DE, USA) until they had fully expired their breath, permitting the sampler to collect alveolar air. We then pushed the collected alveolar air through Markes Carbograph 2TD/Carbograph 1TD thermal desorption tubes using a plunger. The samples were analyzed for aromatic hydrocarbons (benzene, toluene, ethyl benzene, xylene, and styrene) and semi-volatile PAHs (naphthalene, anthracene, phenanthrene, fluoranthene, and pyrene) using a gas chromatography/mass spectrometry (GC/MS) method described in Sobus *et al.* (2008).

### Urine sampling

We assessed systemic PAH and benzene exposure of participants by measuring their PAH and benzene metabolite levels in urine. We also measured urinary creatinine and cotinine. Creatinine was used to normalize the PAH and benzene metabolite results. Urine samples were collected pre-, post-, 3-h, and 6-h post-exposure. The participants were given sterile 100-ml collection cups for their specimens. All urine samples were aliquoted into labeled tubes and stored on dry ice while in the field. On arrival in the lab, samples were stored at −20°C for those pending PAH analysis and at −80°C for those pending cotinine and creatinine analyses.

The urinary PAH-metabolite assay was performed using a modified version of a commercial ELISA (PAH RaPID Assay®, Strategic Diagnostics Inc., Newark, DE, USA) to detect polycyclic aromatic compounds in aqueous samples (Smith *et al.*, 2011). Urine samples diluted 25% with methanol at collection were treated with the enzyme β-glucuronidase to cleave glucuronide conjugates of PAH metabolites followed by 1/20 dilution with kit diluent to diminish urine matrix effects and then assayed according to the instructions. The concentrations were reported as phenanthrene kit equivalents corrected for a dilution factor of 28.8. This method has been shown to correlate well (*r* = 0.89) with the sum of eight PAH metabolites measured by GC/MS in exposed workers’ urine (Smith *et al.*, 2011). Although it may be considered a broad screening tool, it is most sensitive toward the six PAHs measured on skin. The assay detection limits for phenanthrene, fluoranthene, benzo[a]pyrene, pyrene, chrysene, and anthracene were 0.93, 0.43, 0.67, 0.27, 0.53, and 0.72ng ml^−1^, respectively.

The urine samples were not analyzed for the benzene metabolite, s-phenylmercapturic acid (s-PMA), until 1–2 years after collection. We added this test to the study after detecting elevated post-exposure concentrations of benzene in breath compared with the pre-exposure concentrations. These samples were analyzed by NMS Labs (Willow Grove, PA, USA) using an internal LC/MS-MS method. Prior to analysis, 1 drop of 12 N hydrochloric acid was added to each 5-ml urine sample aliquot.

Creatinine was measured using a Vitros Autoanalyzer (Johnson & Johnson, New Brunswick, NJ, USA) with a Vitros CREA slide. Cotinine, a metabolite of nicotine, was measured using Diagnostic Products Corporation (Siemens Corporation, Washington, DC, USA) Immulite® 2000 analytical platform. Analysis of cotinine concentrations was used to confirm current non-smoking status of the participants and to quantify possible exposure to environmental tobacco smoke (Suwan-ampai *et al.*, 2009).

### Statistical analysis

SAS 9.3 statistical software (SAS Institute, Cary, NC, USA) was used for statistical analysis. Because of varying parameters of the two rounds, round 1 and round 2 data were analyzed separately. Non-detectable (ND) measurements were assigned values of the minimum detectable concentration (MDC) divided by the square root of two. MDCs are the lowest concentrations of a particular analyte that can be detected with a sampling method. We used the average volume of air sampled (or average other denominator) to calculate the MDCs. All personal air and breath samples measured detectable levels of PAHs and benzene. However, several wipe samples measured ND levels of PAHs. Of particular interest in this article, 10 of 30 post-exposure wipe samples of the neck measured ND levels for all six PAHs and 5 of 30 urine samples (3-h post-exposure) measured ND levels of PAH metabolites. The MDCs for PAH levels on the neck varied slightly for each of the six PAHs, ranging from 7 to 14 µg m^−2^ in round 1 (57 µg m^−2^ total) and from 7 to 21 µg m^−2^ in round 2 (76 µg m^−2^ total). The MDC for PAH metabolites in urine was 22 µg g^−1^ creatinine. A full list of MDCs is provided in the NIOSH report (NIOSH, 2013b).

We excluded a few personal exposure data from the statistical analyses. The pre-exposure level of PAHs on the neck of one subject (380 µg m^−2^) was excluded from analyses because it was well above all other pre and even post-exposure measurements. One pre-exposure breath measurement of benzene (378 µg m^−3^) was excluded from statistical analysis because we had reason to believe the thermal desorption tube used to make this measurement had not been thoroughly cleaned from prior use. Three air samples were excluded because the tubing became disconnected (as mentioned previously).

New variables representing the change in dermal exposure, urine, and exhaled breath levels over different collection time periods were generated. The distributions of these and other variables were explored. Because the assumption of normality was not reasonable for many of our paired comparisons, non-parametric sign tests were used to explore possible significant changes. Spearman correlations were used to explore relationships between exposure and biomarker variables.

## RESULTS

### External exposures

The personal air concentrations of PAHs are summarized in Fig. 2. Because the firefighters wore SCBA, these do not represent inhalation exposures. The air concentrations of PAHs appear higher and more variable during round 1 than round 2. Of the PAHs measured in air, >95% were in the particulate phase or adsorbed to particles. Table 2 provides the average proportion of known, probably, and possibly carcinogenic PAHs (according to IARC), and other PAHs collected on the personal air samples for each burn. These proportions did not vary substantially within a round (coefficient of variations < 38%). Overall, the round-1 burns produced a greater proportion of ‘potentially’ carcinogenic PAHs than the round-2 burns.

**Table 2.** Average proportion (%) of known, probably, and possibly carcinogenic PAHs and other PAHs collected on personal air samples for each burn

	Round 1			Round 2		
	Burn 1	Burn 2	Burn 3	Burn 1	Burn 2	Burn 3
Benzo[a]pyrene (carcinogenic)	5.7	5.8	6.1	1.6	1.9	2.4
Dibenzo[a,h]anthracene (probably carcinogenic)	3.3	1.4	0.8	2.6	1.2	2.4
Possibly carcinogenic PAHs^a^	39	43	30	30	25	21
Other PAHs^b^	52	50	63	66	72	74

^a^Includes benz[a]anthracene, benzo[b]fluoranthene, benzo[j]fluoranthene, benzo[k]fluoranthene, chrysene, indeno[1,2,3-c,d]pyrene, and naphthalene.

^b^Includes acenaphthene, acenaphthylene, anthracene, benzo[e]pyrene, benzo[g,h,i]perylene, fluoranthene, fluorine, phenanthrene, and pyrene.

**2:**
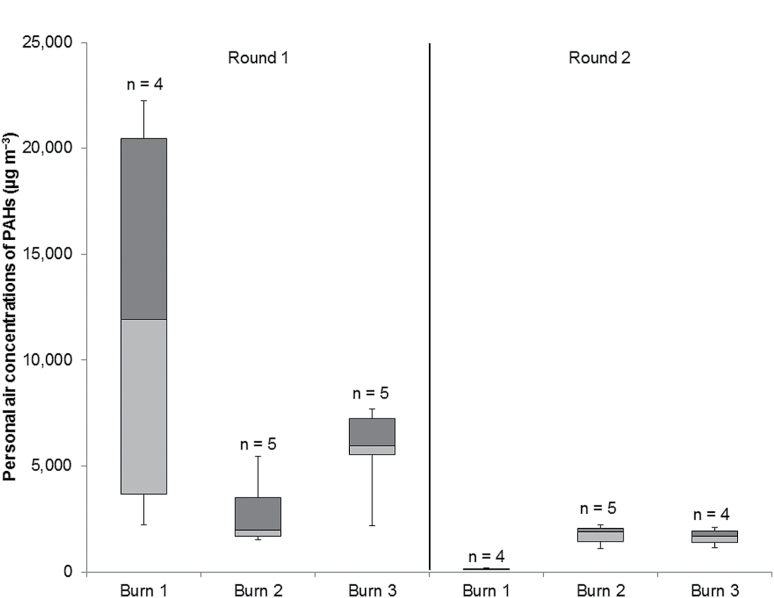
Box and whisker chart showing the personal air concentrations of total PAHs measured during the controlled burns by round.

Table 3 provides the PAH dermal exposure results by skin site. The number of detectable levels on the arm, neck, and scrotum was generally greater in round 2 than in round 1. All median levels were below the sum of MDCs for the 6 PAHs, except for the exposures on the arm in round 2.

**Table 3.** Dermal exposure to PAHs by body site

	Pre-exposure				Post-exposure				Sum of MDCs
	*N*	No. of NDs^a^	Results (µg m^−2^)^b^		*N*	No. of NDs^a^	Results (µg m^−2^)^b^		
			Median	Range			Median	Range	
Round 1									
Arm	15	12	11.5	11.5–14.1	15	8	11.5	11.5–17.4	16
Neck^c^	15	14	40.4	40.4–43.2	15	6	52.0	40.4–187	57
Scrotum	15	14	31.4	31.4–68.0	15	11	31.4	31.4–90.2	44
Hand	0	—	—	—	15	11	15.9	15.9–23.5	22
Face	0	—	—	—	15	9	25.0	25.0–40.8	35
Round 2									
Arm	15	2	21.3	15.3–85.2	15	2	25.2	15.3–92.7	22
Neck	14	8	53.8	53.8–125	15	4	62.8	53.8–160	76
Scrotum	15	7	46.1	42.4–93.4	15	4	50.4	42.4–69.3	60
Hand	0	—	—	—	15	2	23.7	21.1–40.2	30
Face	0	—	—	—	15	3	37.5	33.3–51.1	47

^a^Sample considered ND if all analytes were below their respective MDCs.

^b^Sum of anthracene, benzo[a]pyrene, chrysene, fluoranthene, phenanthrene, and pyrene results. Censored data were assigned values of the MDC divided by square root of 2. Minimum levels are the sum of the assigned values.

^c^Statistically significant difference (*P* < 0.05) between pre- and post-exposure measurements.

The neck was the only skin site where we measured a statistically significant pre- to post-exposure increase in PAH levels (for round 1 only). During round 2, the greatest pre- to post-exposure increase in median PAH levels was measured on the neck, but the difference in PAH levels was not statistically significant (*P* = 0.07). The neck also had the greatest range of post-exposure values for both rounds. For these reasons, subsequent data analyses involving dermal exposure data were focused on PAH levels measured on the neck. Phenanthrene was detected on the neck post-exposure in more than twice the number of samples (*n* = 15) of any other analyte.

Because the corn oil provided a complex matrix, we explored the contribution of the corn oil matrix to the variability in the PAH levels measured on the neck. Analysis of the field blanks (wipe plus corn oil) showed standard deviations of 16 µg m^−2^ (*n* = 6) and 6.5 µg m^−2^ (*n* = 9) for rounds 1 and 2, respectively. This analysis suggests that the corn oil matrix could account for 22–39% of the variability in the PAH measurements on the neck, with the exception of the round 1 pre-exposure measurements that had much less variability (standard deviation = 0.72) than the field blanks. Low pre-exposure variability was not unexpected because the firefighters were instructed to avoid sources of PAHs prior to starting the study.

### Biomarkers

Of the compounds measured in breath, we focus on benzene for this article. This is because benzene represented the predominant aromatic hydrocarbon measured post-exposure in breath with the largest change in median concentrations over time. For both rounds, the median breath concentrations of benzene were elevated post-exposure and subsequently decreased at the 6-h collection. Although not shown here, this trend was repeated for most other aromatic hydrocarbons measured in breath. Other than for naphthalene, this trend was not observed for the semi-volatile PAHs (NIOSH, 2013b).

Figure 3 shows the individual benzene breath concentrations across time. Most round 1 participants (12 of 14) exhibited a post-exposure increase (*P* = 0.01) in exhaled breath concentrations, spanning up to two orders of magnitude. Most round 2 participants (12 of 15) also exhibited a post-exposure increase (*P* = 0.04), spanning up to one order of magnitude. For round 1, nine participants had higher 6-h breath concentrations than their post-exposure levels (versus three in round 2), including seven with benzene ≥ 290 µg m^−3^ (connected by dotted lines in Fig. 3). Tobacco smoke did not appear to be a cause of these elevated 6-h measurements because 6 of 7 of these participants had ND levels of cotinine in their urine.

**3:**
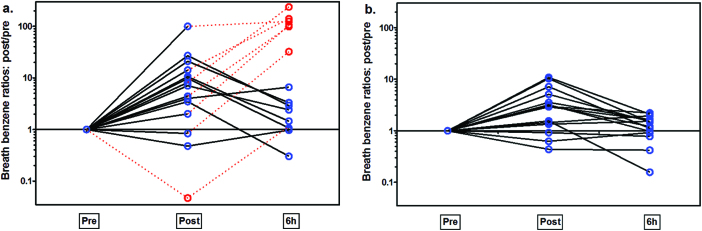
Breath concentrations of benzene wherein the post-exposure and 6-h post-exposure data were normalized to the pre-exposure data at the individual level for round 1 (a) and round 2 (b). Suspect data are connected to other data by dotted lines. This includes the measurement from the participant with the high pre-exposure level and the group of seven 6-h exposure measurements ≥ 290 µg m^−3^ discussed previously.

We ascribe these elevated measurements to a true effect possibly from some uncontrolled post-firefighting benzene exposure. For example, gaseous contaminants produced by training fires that took place later in the day in round 1 (several hours after the exposure period) could have been drawn through the air intake of the building where firefighter participants were stationed. Additionally, the participants might have gone outdoors unbeknownst to us. Such exposure would have mainly affected the 6-h exhaled breath levels of aromatic hydrocarbons and possibly the 6-h urinary PAH levels. This bimodal trait in benzene in breath was not observed in round 2 where no concurrent training exercises took place and where we ensured participants remained indoors after the controlled burns. Figure 3 also includes the high pre-exposure breath concentration (380 µg m^−3^) measured in round 1 that was excluded from statistical analyses. Including this data point resulted in a sharp decrease in post-exposure breath concentrations of benzene for one individual (Fig. 3).

Although we found statistically significant post-exposure increases of benzene in breath, all the urine concentrations of s-PMA were below the LOD of 5 µg l^−1^. After correcting by the average creatinine concentration, the MDC was 8.5 µg g^−1^. Therefore, on average, the systemic exposure to benzene was below the American Conference of Governmental Industrial Hygienists (ACGIH®) Biological Exposure Index (BEI®) of 25 µg g^−1^ creatinine (ACGIH, 2013). This suggests that although some biological uptake occurred (as shown in the breath samples), the total dose of benzene over the short exposure period (≤30min) was not enough to increase urinary excretion of benzene metabolites above exposure criteria.

Figure 4 summarizes the urinary PAH metabolite levels over time. Median urinary PAH metabolite levels appear higher during round 1 than round 2. The highest median urinary PAH metabolite levels were measured during the 3-h collection for both rounds, but the temporal pattern varied between rounds. The PAH metabolite levels in the 3-h samples did not differ significantly from the pre-exposure levels for either round.

**4:**
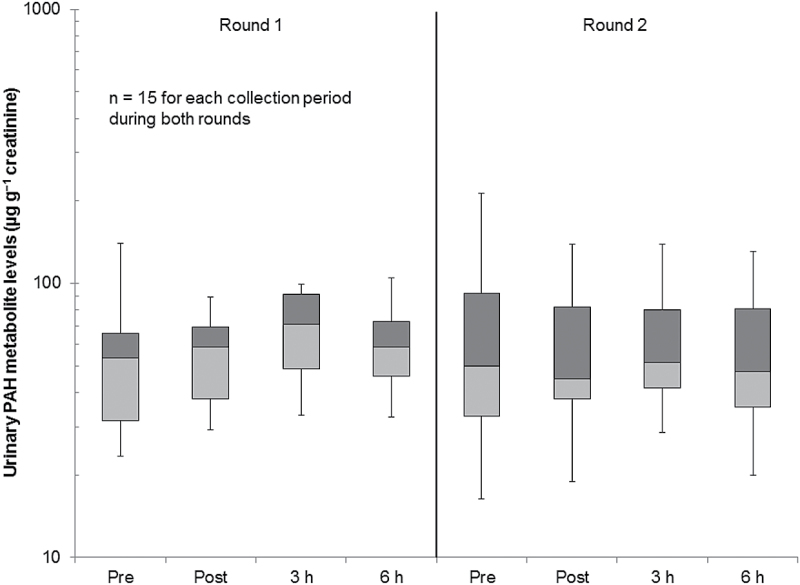
Box and whisker chart showing urinary PAH metabolite levels during different collection periods by round.

Urinary cotinine concentrations above 30 µg l^−1^ may indicate passive or active light cigarette smoking (Wall *et al.* 1988). The majority of the firefighters (23 of 30) had urinary cotinine concentrations below 30 µg l^−1^. Of the seven firefighters with elevated levels, two had urinary cotinine concentrations ranging from ND (<MDC of 10 µg l^−1^) to 66 µg l^−1^ (with levels above 30 µg l^−1^ measured only in the first two collection periods) and five had urinary cotinine concentrations ranging from 45 to 990 µg l^−1^.

### Relationships between external exposures and biomarkers

To explore relationships between external exposures and biomarkers, we created three variables that generally represented the greatest increase in median exposures from one collection point (e.g. pre-exposure) to another collection point (e.g. post-exposure). Table 4 presents the median values of these three variables and the personal air concentrations of PAHs by burn. The firefighters participating in the two burns with the highest median personal air concentrations of PAHs (burns 1 and 3 of round 1) also had the highest median levels of the other exposure and biomarker variables. It is also interesting to note that a ranking of the change in PAH levels on the neck from lowest to highest corresponds with the same ranking of the change in urinary PAH metabolite levels.

**Table 4.** Median values of the four variables by burn that we selected to explore further

Round	Burn	Personal air concentrations of PAHs (µg m^−3^)	Change (post versus pre) in breath concentrations of benzene (µg m^−3^)	Change (post versus pre) in PAH levels on the neck (µg m^−2^)	Change (3h versus pre) in urinary PAH metabolite levels (µg g^−1^)
1	1	11 900	48.1	28.9	17.3
	2	1980	2.81	0.0	−6.48
	3	5970	39.2	47.0	29.3
2	1	131	−0.33	13.4	−2.22
	2	1900	7.39	−17.7	−15.7
	3	1700	18.8	18.5	11.6

Table 5 provides the descriptive statistics for these four variables by round. We saw statistically significant increases in the change in PAH levels on the neck for round 1 and the change in breath concentrations of benzene for both rounds. Although we had 15 or less measurements per variable per round, we explored the correlations among these four variables (Table 6). We chose a sign test *P* value < 0.15 as the inclusion criterion for the correlation analysis. Although benzene has one ring and PAHs have multiple rings, we explored correlations between the benzene and PAH variables because other firefighter studies have shown that emissions of benzene increase with increasing emissions of PAHs (QFRS, 2011a,b). We found two significant correlations; personal air concentrations of PAHs were significantly correlated with the change in urinary PAH metabolite levels in round 1 and with the change in breath concentrations of benzene in round 2.

**Table 5.** Descriptive statistics for the four variables that we selected to explore further

Variables	Units	Round	*n*	ND^a^	Median	Minimum	Maximum	*P* value^b^
Personal air concentrations of PAHs	µg m^−3^	1	14	0	5300	1500	22 000	NA
		2	13	0	1400	130	2200	NA
Change in PAH levels on the neck (post versus pre)	µg m^−2^	1	15	5	12	−2.8	150	0.02
		2	14	3	11	−38	61	0.07
Change in breath concentrations of benzene (post versus pre)	µg m^−3^	1	14	0	34	−11	340	0.01
		2	15	0	7.4	−10	29	0.04
Change in urinary PAH metabolite levels (3h versus pre)	µg g^−1^	1	15	1	17	−61	53	0.12
		2	15	1	−2.1	−100	67	>0.99

^a^Value counted as ND if all analytical data used to calculate the value were ND.

^b^Sign test was used to test if the number of positive differences was significantly different from the number of negative differences.

**Table 6.** Correlations among the four variables

Outcome variable	Explanatory variable	Round	No. of samples	Spearman	
				*r*	*P* value
Change in urinary PAH metabolite levels (3h versus pre)	Personal air concentrations of PAHs	1	14	0.74	<0.01
Change in urinary PAH metabolite levels (3h versus pre)	Change in PAH levels on the neck (post versus pre)	1	15	0.43	0.11
Change in urinary PAH metabolite levels (3h versus pre)	Change in breath concentrations of benzene (post versus pre)	1	14	0.44	0.12
Change in breath concentrations of benzene (post versus pre)	Personal air concentrations of PAHs	1	13	0.36	0.22
		2	13	0.72	<0.01
Change in breath concentrations of benzene (post versus pre)	Change in PAH levels on the neck (post versus pre)	1	14	0.24	0.41
		2	14	0.06	0.84
Change in PAH levels on the neck (post versus pre)	Personal air concentrations of PAHs	1	14	0.48	0.08
		2	12	−0.01	0.97

## DISCUSSION

We evaluated body burdens of PAHs and other aromatic hydrocarbons among firefighters wearing full protective ensembles during live-fire training. Because of the study conditions and requirements, we believe that our findings mainly reflect dermal exposure to airborne combustion byproducts. However, interpretation of the results requires consideration of the small sample size (limited statistical power), potential for additional routes of exposure, and other limitations.

Most of the personal air sampling pumps faulted before completion of overhaul. Thus, personal air concentrations were calculated in a manner to provide an estimate of the air concentrations during the most contaminated active fire period of the response. Area air monitoring results reported elsewhere (NIOSH, 2013b) support our assumption that PAH air concentrations during active fire were several factors higher than after knockdown, but not necessarily our assumption that these air concentrations were fairly constant. Thus, our calculation of air concentrations could have introduced some bias. In addition, the temperature of the air that was sampled exceeded the upper operating range (45°C) of the sampling pumps, which could have altered the suction efficiency and biased the results. Although the direction and magnitude of these biases cannot be determined, we suspect that any bias would be less than the overall variability in the air concentrations.

The firefighters’ personal air concentrations of PAHs (130–22 000 µg m^−3^) were comparable or higher than those measured in other firefighter exposure studies (<5–15 000 µg m^−3^) (Jankovic *et al.*, 1991; Feunekes *et al.*, 1997; QFRS, 2011a,b). Variability in PAH air concentrations may be explained by differences in sampling methods, specific chemical composition of the fuel packages, fire temperatures, compartment size, and ventilation conditions. Some of these factors may have caused the personal air concentrations in round 1 to be higher and more variable than in round 2. In general, the smoke in round 1 appeared to be darker suggesting that these fires may have been more oxygen deprived. Additionally, the firefighters in round 2 conducted simulated firefighting activities, mostly below the smoke layer, whereas firefighters in round 1 passively viewed the fire, standing for portions of the exposure period. Of particular note, two firefighters from burn 1 of round 1, who stood toward the back of the structure where there was less air movement, had substantially higher exposures (19 000 and 22 000 µg m^−3^) than the rest of their crew (2200 and 5100 µg m^−3^).

We believe that we obtained a reasonable estimate of dermal exposure by summing individual PAHs that were not affected by the corn oil matrix. The urine ELISA method was most sensitive toward the six PAHs that were included in the dermal exposure variable, so exploring relationships between dermal exposures and urinary excretion of PAHs should be valid. The dermal exposure data suggest that the neck skin was the most exposed part of the firefighters’ bodies. Unlike the other areas of the firefighters’ bodies that were covered in multiple layers (including a semipermeable moisture barrier) of protective clothing, the neck was primarily protected by a Nomex® hood made of a double layer of porous flame-resistant fabric. The shorter hoods used in round 1 could have further increased the potential for dermal exposure to the neck and may partially explain why the post-exposure increase of PAHs on the neck was significant for round 1 (*P* = 0.02) but not round 2 (*P* = 0.07). However, the results are similar for both rounds and support the need for further research on the design of hoods to minimize chemical permeation and penetration and reduce the potential for hoods becoming untucked during a response.

Although benzene was elevated in the post-exposure breath of the firefighters, urinary excretion of s-PMA was below the MDC of 8.5 µg g^−1^ for all samples. The s-PMA analysis of urine did not occur until as much as 2 years after collection. Because the urine samples were stored in a −20°C freezer, it is unlikely that the benzene metabolite degraded. Thus, our data suggest that the overall dose of benzene resulted in urinary s-PMA levels below the ACGIH® BEI® of 25 µg g^−1^ creatinine (ACGIH, 2013).

The benzene in breath and urinary PAH metabolite levels we measured are comparable to the levels measured using the same types of methods in low exposed employee populations. The post-exposure breath concentrations of benzene in the participating firefighters (median = 19 µg m^−3^, range = 3.3–350 µg m^−3^) were nearly equivalent to the post-exposure breath concentrations of benzene in non-smoking automobile mechanics after 4h of work (median = 19 µg m^−3^, range = 3.5–500 µg m^−3^) (Egeghy *et al.*, 2002) and comparable to exhaled breath concentrations measured in U.S. Air Force fuel system maintenance workers and operational ground crews (means ranging from 1.9 to 50 µg m^−3^) (Pleil *et al.*, 2000; Pleil, 2009; Pleil *et al.*, 2011). The 3-h urinary levels of PAH metabolites in the participating firefighters (median = 62 µg g^−1^, range = 29–140 µg g^−1^) were within the range of baseline PAH metabolites in non-smoking asphalt pavers (median = 110 µg g^−1^, range = 57–140 µg g^−1^) (McClean *et al.*, 2012) and non-smokers without occupational PAH exposures (median = 67 µg g^−1^, range = 6–220 µg g^−1^) (Osborn *et al.*, 2011; Smith *et al.*, 2011). Note that the firefighters may have come to the study with lower than normal levels of PAHs in their system due to our pre-study requirements, while the comparison populations above did not have these requirements.

We found statistically significant positive correlations between external exposures (i.e. personal air concentrations of PAHs) and biomarkers (i.e. change in urinary PAH metabolite levels in round 1 and change in breath concentrations of benzene in round 2). The latter relationship may be explained by PAHs and benzene both being products of combustion and, hence, interrelated (QFRS, 2011a,b). Other correlations were not statistically significant. Certainly, the weak statistical power could have played a role in some of the null findings. In addition, the differences in the strengths of the correlations between rounds may be due in part to the lower and less variable airborne PAH concentrations produced in round 2, which could have led to lower body burden. This is evident in Table 4 as the lowest median air concentrations of PAHs were all measured in round 2. Another interesting finding shown in this table was that the median change in PAH levels on the neck appears to be associated with the median change in urinary PAH metabolite levels by burn.

All together, the data suggest that some PAHs and benzene generated by the fires were absorbed into the firefighters’ bodies. One of three exposure pathways may have led to the uptake of external exposures. First, firefighters could have overbreathed their SCBA. Burgess and Crutchfield (1995) demonstrated this phenomenon in firefighters under high physical exertion (walking on an inclined treadmill) with high breathing rates (90–160 l min^−1^, 17–36 breaths min^−1^) although they still found protection factors > 10 000. The tasks performed by firefighters in our study likely produced exertion rates less than those in Burgess and Crutchfield (1999). In addition, we tested the SCBA used in round 2 and found that all but one passed the manikin breathing tests (including tests for positive pressure) at maximum respiration (103±3 l min^−1^, 30±1 breaths min^−1^) (NIOSH, 2013b). This maximum respiration rate is at the upper range of the breathing rates in Burgess and Crutchfield (1995). For these reasons, we believe the firefighters did not overbreathe their SCBA. Second, firefighters could have inhaled substances evaporating from their contaminated clothing and equipment when they were doffing their gear. Low concentrations of aromatic hydrocarbons (generally < 100 µg m^−3^) have been shown to off-gas from contaminated gear several minutes after a response (NIOSH, 2013b). Doffing took ~2–4min, so any inhalation exposures to benzene during the doffing of gear were of short duration. Unlike aromatic hydrocarbons, most PAHs are semi-volatile or non-volatile at ambient temperatures. Thus, evaporation from contaminated gear is an unlikely exposure pathway for PAHs. Third, firefighters could have dermally absorbed PAHs and benzene present in air during the controlled burns. We believe this is the most plausible exposure pathway for the PAHs and benzene, and that the neck was the main site of exposure and absorption based on our dermal wipe results.

Several studies have measured dermal absorption of PAHs (Storer *et al.*, 1984; Kao *et al.*, 1985; VanRooij *et al.*, 1993; Roy *et al.*, 1998) and benzene vapor (Maibach and Anjo, 1981; Franz, 1984; Thrall *et al.*, 2000; Wester and Maibach, 2000). VanRooij *et al.* (1993) found that 20–56% of PAHs (as a low dose of coal tar) on the skin will be absorbed within 6h depending on the anatomical site of the dose. In general, anatomical sites with thinner skin (e.g. neck) had faster absorption rates (VanRooij *et al.*, 1993). Humidity and sweat are important factors for dermal absorption of benzene. Franz (1984) discovered that dermal absorption of benzene vapor was 2.5–7.5 times greater in 100% relative humidity environments than 40% relative humidity environments. Franz (1984) also reported that 5–6% of the applied dose was absorbed when benzene was dissolved in water versus <0.20% when it was dissolved in toluene. Thrall *et al.* (2000) measured peak concentrations of benzene in breath ~2h after topical application of benzene (dissolved in water) to monkey skin (*in vivo*). Elevated surface temperature of skin may also increase dermal absorption due to increased surface blood flow and opening of skin pores (Jones *et al.*, 2003).

Our findings are similar to the findings in other studies where investigators measured biomarkers of PAHs and benzene in firefighters who wore SCBA the majority of the time during controlled (Laitinen *et al.*, 2010) and uncontrolled fires (Caux *et al.*, 2002). Caux *et al.* (2002) found that the largest increase in urinary excretion of 1-hydroxypyrene occurred between 4 and 8h after the exposure period but were unable to identify the peak excretion of muconic acid due to relatively minor changes. Laitinen *et al.* (2010) found that the largest increase in 1-hydroxypyrene and 1-napthol occurred 6h after the exposure period and the largest increase in muconic acid occurred immediately after the exposure period. The exposure period in Laitinen *et al.* (2010) was ~3 times longer than our exposure period, which could explain why they detected an increase in a urinary benzene metabolite, but we did not. The absorption and excretion rates of PAHs in our study appear slightly faster than these studies. This could be due to inter-study differences in composition of PAHs produced, the primary site of dermal exposure, duration of exposure, and environmental conditions of the fires.

Animal studies have shown that exposure to PAHs can cause various types of cancer, most often at the site of dosage, but occasionally at more distant sites (American Conference of Governmental Industrial Hygienists [ATSDR], 1995; IARC, 2002 , 2010). Occupational epidemiology studies have primarily found associations between exposures to PAHs (typically as a mixture with other chemicals) and lung, skin, or bladder cancer, depending on the route of exposure (ATSDR, 1995; Boffetta *et al.*, 1997; IARC, 2002 , 2010). Occupational exposure to benzene has been consistently linked to leukemia (IARC, 2012; ATSDR, 2007). Because PAH exposure to the scrotum has been associated with scrotal cancer in chimney sweeps (Hall, 1998), the possibility exists that PAH exposure to the scrotum could also lead to testicular cancer in firefighters. However, we did not find significantly elevated post-exposure levels of PAHs on the scrotum. It is important to note that the NFPA 1971/1981 compliant full protective ensembles for structure fires worn by firefighters in this study likely provides more protection to the groin area than the traditional long coats and 3/4 boots that were worn by many firefighters in the past.

Some studies have found elevated risk for the aforementioned cancers in firefighters, whereas others have not. In a meta-analysis of cancer studies in firefighters, LeMasters *et al.* (2006) found a probable or possible increased risk for 12 types of cancer, including leukemia, skin, and testicular cancer, but not lung or bladder cancer. More recently in the largest firefighter cancer study to date, Daniels *et al.* (2013) found that firefighters had an increased incidence of eight types of cancers, including lung and kidney cancer, but not leukemia, skin, or testicular cancer. More research is needed to quantify and better understand these cancer risks and the role that chemical exposures may play.

## CONCLUSIONS

We found that firefighters wearing full protective ensembles can have systemic exposures to PAHs and other aromatic hydrocarbons. Our results show that PAHs most likely entered the firefighters’ bodies through their skin, with the neck being a primary site of exposure and absorption due to the lower level of dermal protection afforded by hoods. Aromatic hydrocarbons could also have been absorbed dermally during firefighting or inhaled during the doffing of gear that was off-gassing contaminants. Although the systemic exposures we measured are comparable to the levels measured in low exposed employee populations, the absorbed dose will depend on the variable air concentrations of PAHs and other aromatic hydrocarbons generated during fires, as well as the total duration of the fire response and adherence in wearing full protective ensembles. Further study on hood design, turnout gear decontamination methods, and firefighters’ dermal absorption of combustion products during fire responses is warranted.

## FUNDING

National Institute for Occupational Safety and Health (NIOSH) by intramural award under the National Occupational Research Agenda; NIOSH Human Subjects Review Board. 
